# Bronchial isomerism in a Kabuki syndrome patient with a novel mutation in *MLL2* gene

**DOI:** 10.1186/1471-2350-15-15

**Published:** 2014-01-28

**Authors:** Gerarda Cappuccio, Alessandro Rossi, Paolo Fontana, Emma Acampora, Valeria Avolio, Giuseppe Merla, Leopoldo Zelante, Aurelio Secinaro, Generoso Andria, Daniela Melis

**Affiliations:** 1 Department of Translational Medical Sciences, Section of Pediatrics, Federico II University, Via Sergio Pansini 5, 80131 Naples, Italy; 2 Medical Genetics Unit, IRCCS Casa Sollievo della Sofferenza, San Giovanni Rotondo, FG, Italy; 3 Bambino Gesù Children Hospital, IRCCS, Rome, Italy

**Keywords:** Kabuki syndrome, Isomerism, Respiratory distress

## Abstract

**Background:**

Kabuki syndrome (KS) is a rare, multiple congenital anomalies/intellectual disability syndrome caused by mutations of *MLL2* gene, which codifies for a histone methyltrasferase that regulates the embryogenesis and the tissue development. Left-bronchial isomerism is a rare congenital abnormality that can be defined as the absence of the normal lateralizing features which distinguish right and left-sides in the lungs. To date, this is the first report of left-bronchial isomerism in association with KS.

**Case presentation:**

A one-month-old Caucasian male patient underwent our attention for microcephaly, dysmorphic features (long palpebral fissures, eyebrows with sparse lateral third, everted lower eyelids, blue sclerae, large dysplastic ears, lower lip pits), persistent fetal fingertip pads, short stature, heart defects (interventricular defect and aortic coarctation), unilateral cryptorchidism, hypotonia and delay in gross motor skills. These features suggested a diagnosis of KS and a molecular analysis confirmed a novel frame-shift mutation in the exon 11 of *MLL2* gene. Subsequently, given recurrent respiratory infections with a normal immunological status, he underwent a chest CT scan that showed a left bronchial isomerism.

**Conclusion:**

We report a patient affected by KS, with a novel *MLL2* mutation and an atypical phenotype characterized by left-side bronchial isomerism. Interestingly, genes involved in the heterotaxia/isomerism such as *ROCK2* and *SHROOM3* are known to interact with *MLL2* gene. In order to achieve a correct diagnosis and an appropriate therapy, the presence of pulmonary anatomical variations should be investigated in KS patients with respiratory signs not associated to immunological deficiency. Finally, our findings support the hypothesis that the mutations leading to a complete loss of function of *MLL2* gene is often associated with complex visceral malformations.

## Background

Kabuki syndrome (KS) is a rare congenital disorder, characterized by typical facial features including: long palpebral fissures, eversion of the lateral third of the lower eyelids, arched and broad eyebrows with lateral sparseness, short columella and large prominent ears. Other main clinical features are: mild or moderate intellectual disability, persistent fetal fingertip pads, minor skeletal anomalies (digital and vertebral anomalies) and hypodontia. Additional features include short stature, internal malformations (involving the heart, genitourinary and gastrointestinal systems) and immunological defects [1].

Point mutations and large intragenic deletions and duplications of the histone methyl transferase *MLL2* gene are the main causes of KS [2-9]. *MLL2* encodes a large protein that belongs to the SET1 family of human SET-domain protein methyltransferase superfamily. Recently, *de novo* partial or complete deletions and point mutations of the gene, lysine demethylase 6A (*KDM6A*), mapping at Xp11.23 have been identified as additional causes of KS confirming the genetic heterogeneity of the disease [10,11]. *KDM6A* demethylates di- and trimethyl-lysine 27 on histone H3, maintaining embryonic stem cell pluripotency and plasticity during embryonic development and X chromosome inactivation [12].

Lateralization disorders are divided into complete (*ie*, situs inversus totalis) and incomplete forms (*ie*, heterotaxy). In particular, heterotaxy is a malformation where the internal thoraco-abdominal organs demonstrate abnormal arrangement across the left-right axis of the body, often involving a wide range of very complex cardiac lesions. Heterotaxy syndromes can be further classified in two main subgroups, including right-sided isomerism and left-sided isomerism, often associated with asplenia and polysplenia, respectively. In isomerism, the absence of the normal lateralizing features makes right and left-sided organs hard to distinguish. Left bronchial isomerism is characterized by anatomical features of bilateral left lung (two bilobed lungs, each one with a long main bronchus) [13].

Here we describe a male patient with a novel 11 nucleotides deletion in exon 11 of *MLL2* gene, presenting with distinctive features of KS and atypical ones such as neonatal hypoglycemia, and left-side bronchial isomerism.

## Case presentation

### Clinical report

The patient was born at 37 weeks of gestation by spontaneous vaginal delivery. The pregnancy was complicated by intrauterine growth restriction during the first trimester; furthermore prenatal ultrasound showed left pyelectasis, single umbilical artery, aortic coarctation with ventricular septal defect. His parents were unrelated and there was no family history of any known inherited condition. His birth weight was 2.420 Kg (<10^th^ centile), length 47 cm (25^th^ centile), head circumference 31 cm (<5^th^ centile). The Apgar scores were 7 at 1’, 8 at 5’. At birth, he was admitted to intensive care unit for the presence of respiratory distress and hypoglycemia (44 mg/dl), quickly reverted after treatment with glucose solution. At 4 days of life he underwent surgery for aortic arch enlargement and coarctation repair through a left thoracotomy.

**Figure 1:**
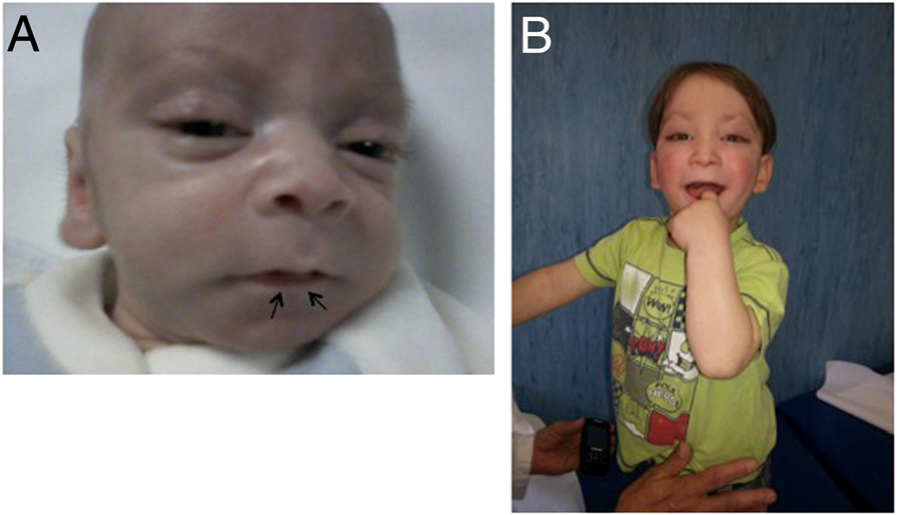
Patient facial appearence at a) 20 days and b) 1 year and 10 months of age respectively; note the high forehead, lateral sparseness of the eyebrows, long palpebral fissures, everted lower eyelids, blue sclerae, large dysplastic ears, lower lip’s pits (see the arrows).

Since he was one-month-old, he was referred to Genetic Pediatric Clinic Unit of the Department of Translational Medicine, Federico II University. The physical examination revealed: microcephaly, peculiar facial appearance (high forehead, lateral sparseness of the eyebrows, long palpebral fissures, everted lower eyelids, blue sclerae, large dysplastic ears, lower lip pits), persistent fetal fingertip pads, bilateral gynecomastia, right cryptorchidism and inguinal hernia and hypotonia. His weight-for-length ratio was significantly below the 5^th^ percentile (Figure 1A, B). An X-ray showed a D6 “butterfly vertebra”. He exhibited a delay in gross motor skills. A clinical diagnosis of KS was proposed. At the age of six months, auxologic parameters were: weight 4,810 kg (<5°); length 62,8 cm (<5°); W/L <5° ct; head circumference 37 cm (<5°). Cryptorchidism was still present.

**Figure 2:**
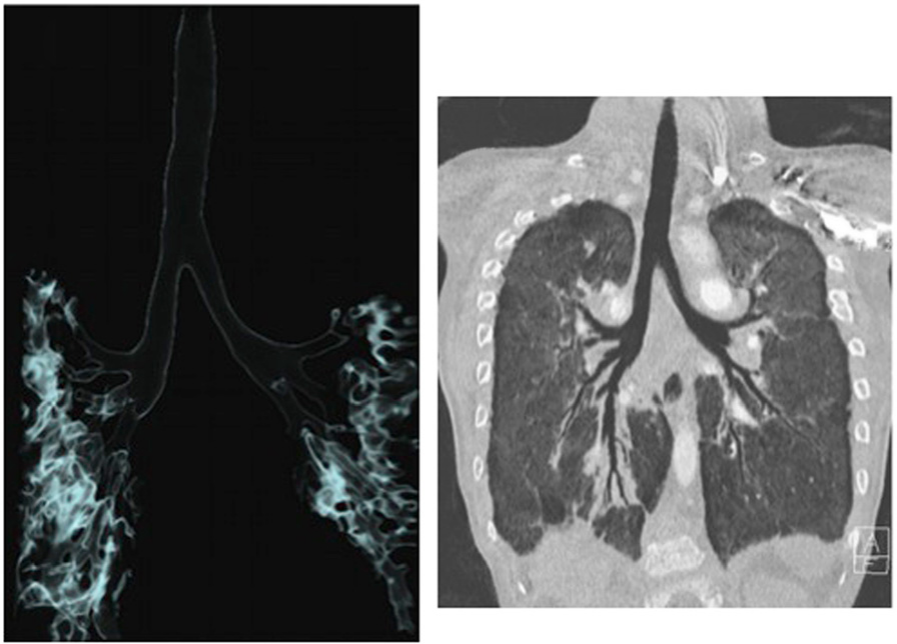
Computed tomography angiography shows long main bronchus, two hyparterial bronchi and bilobed lungs (consistent with left bronchial isomerism), several consolidative opacities, widespread peripheral cylindrical bronchiectasies, bronchial dilatation and bronchial wall thickening.

Transfontanellar ultrasound showed corpus callosum dysgenesis; ophthalmologic examination identified pale optic disc in the right eye. Abdominal ultrasound scan was normal; no asplenia or polysplenia were observed. Two other episodes of hypoglycemia (blood glucose levels 44 and 55 mg/dl, respectively) occurred in the first months of life, both required intravenous dextrose. Since he suffered from recurrent pneumonia, an immunologic evaluation was performed including: levels of total Ig and IgG subclasses, lymphocyte classes, lymphocyte proliferation assays and specific antibody responses, showing normal data. At the age of 1 year, a chest CT scan with iv injection of iodine contrast agent was performed, showing left-side bronchial isomerism (long main bronchus, two hyparterial bronchi and bilobed lungs), several consolidative opacities in the upper and lower lobes in the right lung, widespread peripheral cylindrical bronchiectasies, bronchial dilatation and bronchial wall thickening. Moreover, overinflations in the apical segment of the upper lobes, hyperdense areas and septal lines in posterior basal segments and diffuse lung architectural distortion, were observed. In addition, Azygos lobe was detected (Figure 2).

### Cytogenetic analysis and molecular MLL2 gene study

Informed consent was obtained prior to performing any cytogenetics and molecular assay. Peripheral blood was collected from the affected child and his parents. High resolution karyotype revealed no abnormalities. Molecular genetic testing for suspected KS was performed by complete DNA sequence analysis of the entire coding region and flanking introns of the *MLL2* gene, as reported [5]. We detected a *de novo* deletion in exon 11 (c.3161_3171delCGTTGAGTCCC, p. Pro1054HisfsX10). The predicted protein lacks the most important functional domains of MLL2, the plant homeodomains (PHD_4-6_) and the SET domains that cooperate with the methyltransferase activity of MLL2 [14]. Thus, although not experimentally investigated in this report, the detected mutation would be predicted to affect the physiological function of MLL2 altering the epigenetic and transcriptional regulatory properties of the protein.

## Discussion

We describe a patient with KS confirmed by molecular study, suffering from recurrent respiratory infections due to left lung anomalies.

The patient carries a 11 bases deletion in the exon 11 of the *MLL2* gene, which consists of 54 exons. This deletion determines a frameshift and a stop codon 10 nucleotides downstream from this site. The mutation product is a truncated protein with highly probable loss of function. However we cannot rule out the possibility that this mutation result in the degradation of partial or total transcripts due to nonsense-mediated mRNA decay (NMD), thus contributing to MLL2 protein haploinsufficiency. This specific deletion has not been previously described, but its effect can be compared with other frameshift and with non-sense mutations. The patient described carrying a truncating mutation of *MLL2* presented a complex heart defect (interventricular septal disorder and aortic coarctation), as previously reported in the literature [3].

KS main features are intellectual disability, post-natal growth delay and peculiar facial appearance. The patient herein described showed microcephaly, dysmorphic features (long palpebral fissures, eyebrows with sparse lateral third, everted lower eyelids, blue sclerae, large dysplastic ears, lower lip pits), persistent fetal fingertip pads, short stature, heart defects (interventricular defect and aortic coarctation) and genitourinary malformation, leading to a score of 6 according to Makrythanasis et al. [9]. In addition, neonatal hypoglycemia, not usually detected in this syndrome, was observed in the patient.

Although respiratory infections are frequent in KS [15], and possibly due to immune system dysfunction, immunological studies showed normal data in our patient. The recurrent and severe bronchitis caused multiple hospitalizations, leading to a worsening of the patient’s life quality. The chest CT scan performed with iv injection of iodine contrast agent showed left bronchial isomerism and several bronchiectasies. Bronchial isomerism is a rare congenital abnormality defined as the absence of the normal lateralizing features which distinguish right and left-sided in the lungs. The morphology of the bronchial tree is defined on the basis of: the length of the main bronchus, the course of pulmonary artery on the bronchus and the number of lobes in the lung [16]. Thus, the left bronchial isomerism is characterized by a long main bronchus, two hyparterial bronchi, two bi-lobed lungs. The atrial arrangement frequently follows that of the bronchial tree [13]. Two patients with KS were diagnosed with an anomalous course of the left pulmonary artery whereas a morphological alteration of the bronchial disposition was not investigated [17,18]. Left isomerism may be associated with malrotation of abdominal viscera, polysplenia and congenital heart disease [16]. In our patient an echocardiogram confirmed normal atrial arrangement and an abdominal ultrasound scan showed normal data with no spleen anomalies. Several patients with bronchial isomerism present with severe respiratory injuries, such as: recurrent episodes of coughing, wheezing, asthma and bronchiectasies. In particular three patients diagnosed with severe asthma, classified as resistant to high-dose corticosteroid therapy, were found to have left bronchial isomerism [13,16,19]. With the detection of left-bronchial isomerism in our patient, in order to prevent his recurrent pulmonary infections, aggressive antibiotic combination together with a regular chest physiotherapy was started, improving his respiratory performances.

Although the genetic basis of heterotaxy remains largely unknown, recently several genes have been recognized in the pathogenesis of this malformation. In particular, *SHROOM3* gene has been associated with heterotaxy syndrome; it encodes a cytoskeleton protein responsible for cellular shape during morphogenesis and it has been proposed as a good candidate for the control of left-right (LR) patterning. One of *SHROOM3* downstream effectors is Rho Kinase 2 (*ROCK2*) involved in the proper specification of LR asymmetry during embryonic development [20]. *ROCK2* pathway mediates several cellular functions such as cell shape modification, migration and proliferation, through the phosphorylation of different actin dependent targets [21,22]. Interestingly *MLL2* has been shown to interact with both *SHROOM3* and *ROCK2* proteins. On the basis of these data we suggest that patients with *MLL2* deficiency and recurrent respiratory infections should be investigated for defects of lateralization.

## Conclusions

We suggest to investigate the presence of bronchial tree anatomical variations in KS patients with recurrent respiratory infections and normal immunological parameters. Because of respiratory infections-related bronchial isomerism are a relevant cause of bronchiectasies, it is important to perform a precocious diagnosis and to consider an appropriate treatment. A multidisciplinary approach could consist of: aggressive medical treatments, flu vaccine and respiratory physiotherapy, whereas surgery should be considered in selected patients with extremely severe bronchiectasies.

In the attempt to state the frequency of lateralization defects in KS and in order to investigate if bronchopulmonary congenital malformations are more frequently associated to KS in presence of non-sense mutations of *MLL2* gene, a study of broncho-pulmonary anatomy should be performed.

## Consent

Written informed consent was obtained from the patient’s parents to take part in the study as well as for publication of the images (including full-face pictures). A copy of the written consent is available for review by the Editor-in-Chief of this journal.

## Competing interests

The authors declare that they have no competing interests.

## Authors’ contributions

GC, AR, PF, EA, VA and DM consulted the family, conceived the manuscript; GM performed molecular testing of the patient; AS performed the lung CT-angiography; DM and GA critically revised the manuscript. All authors read and approved the final manuscript.

## Pre-publication history

The pre-publication history for this paper can be accessed here:

http://www.biomedcentral.com/1471-2350/15/15/prepub
